# Cohort Profile Update: The Harmonised Cognitive Assessment Protocol sub-study of the Northern Ireland Cohort for the Longitudinal Study of Ageing (NICOLA-HCAP)

**DOI:** 10.1093/ije/dyag029

**Published:** 2026-03-05

**Authors:** Calum Marr, Leeanne O’Hara, Nicola A Ward, Stanley Simoes, Michael McAlinden, Charlotte Sterling, Claire Potter, David R Weir, Bernadette McGuinness

**Affiliations:** Centre for Public Health, Queen’s University Belfast, Belfast, United Kingdom; Centre for Public Health, Queen’s University Belfast, Belfast, United Kingdom; Centre for Public Health, Queen’s University Belfast, Belfast, United Kingdom; Centre for Public Health, Queen’s University Belfast, Belfast, United Kingdom; Centre for Public Health, Queen’s University Belfast, Belfast, United Kingdom; Centre for Public Health, Queen’s University Belfast, Belfast, United Kingdom; Centre for Public Health, Queen’s University Belfast, Belfast, United Kingdom; Institute for Social Research, University of Michigan, Ann Arbor, MI, United States; Centre for Public Health, Queen’s University Belfast, Belfast, United Kingdom

**Keywords:** NICOLA, Harmonised Cognitive Assessment Protocol, prevalence, cross-national harmonization, longitudinal, ageing, cognition, dementia, cohort, older adult

Key Features

- The Northern Ireland Cohort for the Longitudinal Study of Ageing (NICOLA) was established to provide data on the health and well-being of older adults in Northern Ireland, with the Harmonised Cognitive Assessment Protocol (HCAP) sub-study designed to collect detailed population-level data on dementia and cognitive impairment.
- Between 2022 and 2024, the NICOLA-HCAP study was carried out, involving a randomly selected subset of 1037 Wave 2 (W2) NICOLA participants aged ≥65 years, as well as 862 informants.
- A comprehensive cognitive battery assessed memory, executive function, language, orientation, and visuospatial ability, enabling the algorithm-based classification of cognitive impairment and dementia harmonized with sister HCAP studies: the US Health and Retirement Study, The Irish Longitudinal Study on Ageing, and the English Longitudinal Study of Aging.
- NICOLA (W1, W2) uniquely provides data across psychosocial (e.g. conflict related to The Troubles), lifestyle (e.g. nutrition/environmental exposures), and biological domains (including microbiome profiling, molecular biomarkers, kidney and eye health, neurodegeneration biomarkers, and multi-omic analyses), with W3 currently in the field.
- Data are obtainable via the NICOLA Data Access Committee (https://www.qub.ac.uk/sites/NICOLA/ForResearchers/) and through public repositories, including the Dementias Platform UK data portal (https://portal.dementiasplatform.uk/) and the Gateway to Global Aging Data platform (https://g2aging.org/home).

## The original cohort

The Northern Ireland Cohort for the Longitudinal Study of Ageing (NICOLA) is an ongoing population-based cohort study focusing on the health and well-being of older adults [1]. The study sample comprises a nationally representative cohort of individuals aged ≥50 years (*n* = 8478) living in Northern Ireland (NI), with two waves of data collection completed to date. Wave 1 (W1) of data collection (2013–16) included a face-to-face computer-assisted personal interview (CAPI), a self-completion questionnaire (SCQ), and an in-person physical health assessment. Of the original sample, 6852 participants (81%) completed Wave 2 (W2) (2017–22), which included repeated CAPI and SCQ components. NICOLA data encompass a wide range of detailed demographic, economic, medical, psychosocial, and lifestyle measures. NICOLA also collects objective physical health measures and biological samples for providing biomarker and multi-omic analysis. Information on data access is provided on the NICOLA study website (https://www.qub.ac.uk/sites/NICOLA/).

Data collected by NICOLA are closely harmonized to those of the English Longitudinal Study of Ageing (ELSA) [2] and The Irish Longitudinal Study of Ageing (TILDA) [3] to facilitate cross-national comparisons. NICOLA is also a member of the family of studies connected to the US-based Health and Retirement Study (HRS) [4], and actively contributes to efforts to enable further cross-country data harmonization via the Gateway to Global Ageing (GGA) Data initiative (https://g2aging.org/home).

## What is the reason for the new data collection?

As the proportion of older adults in the global population continues to increase [5], the prevalence of dementia is also projected to increase [6]. Dementia refers to changes in memory and thinking that are driven by neurodegenerative diseases, predominantly Alzheimer’s disease. Dementia symptoms typically progress until an individual’s ability to carry out everyday activities is impaired. Mild cognitive impairment (MCI) is a condition in which individuals have a moderate impairment in memory or thinking without functional impairment; there is an increased risk of developing dementia [7]. Loss of independence can have an impact both at the individual level, due to feelings of loss, stigmatization, and decreased quality of life [8, 9], and at the societal level, due to the growing cost of providing care. The total costs of dementia care in NI have been projected to rise from £0.8 billion in 2019 to £2.4 billion in 2040 [10].

Accurate estimates of the prevalence of dementia and MCI are vital for future care planning and policy development. A recent study estimated that, globally, 57.4 million people were living with dementia in 2019—a number projected to rise to 152.8 million by 2050 [6]. However, there is some variation in the prevalence rates between countries [11] and almost two-thirds of dementia cases remain undiagnosed globally, with rates ranging from 31% to 96%, depending on the region and setting [12]. Representative population-based cohort studies such as NICOLA are an invaluable tool for investigating cross-national variations in dementia prevalence at the population level. The Harmonised Cognitive Assessment Protocol (HCAP) aimed to facilitate detailed cognitive assessments that are directly comparable across the range of international cohort studies centred on HRS. HCAP is a flexible and comparable research instrument for population-level assessment, but it is not intended for clinical diagnosis. HCAP comprises a battery of neuropsychological tests administered to study participants and an informant interview with a participant’s family member or friend. Initially designed and administered as a sub-study within HRS, the protocol has since been implemented in a growing network of at least nine distinct studies worldwide [13–18]. By harmonizing approaches at the design, data-collection, and analysis stages, the HCAP network is creating an international data resource that is useful for comparing dementia prevalence rates across countries. Drawing on the vast amount of data already collected in previous waves of the core studies (including physical health measures, psychosocial and lifestyle measures, and linked administrative data), the HCAP data resource will also allow the cross-national investigation of risk factors for dementia and cognitive decline.

## What will be the new areas of research?

The NICOLA-HCAP sub-study collected cognitive data from a sub-sample of NICOLA participants aged ≥65 years. Data collected will be used to establish the research diagnoses of dementia or MCI among the sub-sample by using an algorithm-based approach already established in HRS-HCAP [19]. Population weights will be developed towards providing the first estimates of dementia and MCI prevalence in the population of NI.

Analyses are planned to utilize data collected in previous waves of the core NICOLA study to examine the potential risk factors for dementia in several areas, including sensory impairment, diet, physical activity, and environmental exposures. The availability of the data on exposure to civil conflict in NICOLA and other international studies in the HCAP network will allow the cross-country examination of the way in which conflict-related stress and trauma may impact cognitive health in older age.

## Who is in the cohort?

The NICOLA-HCAP sub-study was conducted following W2 of the core study and aimed to recruit 1000 participants from the existing cohort, ensuring comparability between the UK and Irish studies (NICOLA, TILDA, ELSA) with other international HCAP studies (e.g. HRS). Participants were eligible for NICOLA-HCAP if they had completed the NICOLA W2 CAPI, were aged ≥65 years on 1 September 2021, and were community-dwelling, consistently with the original NICOLA sampling frame and other longitudinal studies in their early waves (e.g. ELSA [14]). The recruitment process involved sending an invitation letter, followed by telephone screening to confirm that the participant met the eligibility criteria. At this point, any participant who reported having a physical or sensory impairment that would interfere with their ability to complete the tests (e.g. significant visual/hearing impairment) was excluded. An appointment was then made for the participant interview.

Fieldwork for NICOLA-HCAP was conducted between 7 February 2022 and 4 December 2023. The protocol for both participant and informant interviews, including consent procedures, has been detailed elsewhere [20]. Participant interviews were conducted in either participants’ homes or a research centre by a clinical research nurse or research assistant. During the interview, participants nominated an informant and provided informant contact details. Of the nominated informants (Supplementary Table S1), over half were spouses/partners (55%), followed by children (29%), siblings (5%), friends/neighbours (7%), and others (4%). If the informant was present and available, then the informant interview was completed in person following the participant interview. If the informant was unavailable, then they were subsequently contacted by the researcher and either completed the interview over the phone or were provided with a link to complete the measures as an online questionnaire using Qualtrics software (https://www.qualtrics.com).

### Response rates

**Figure 1:**
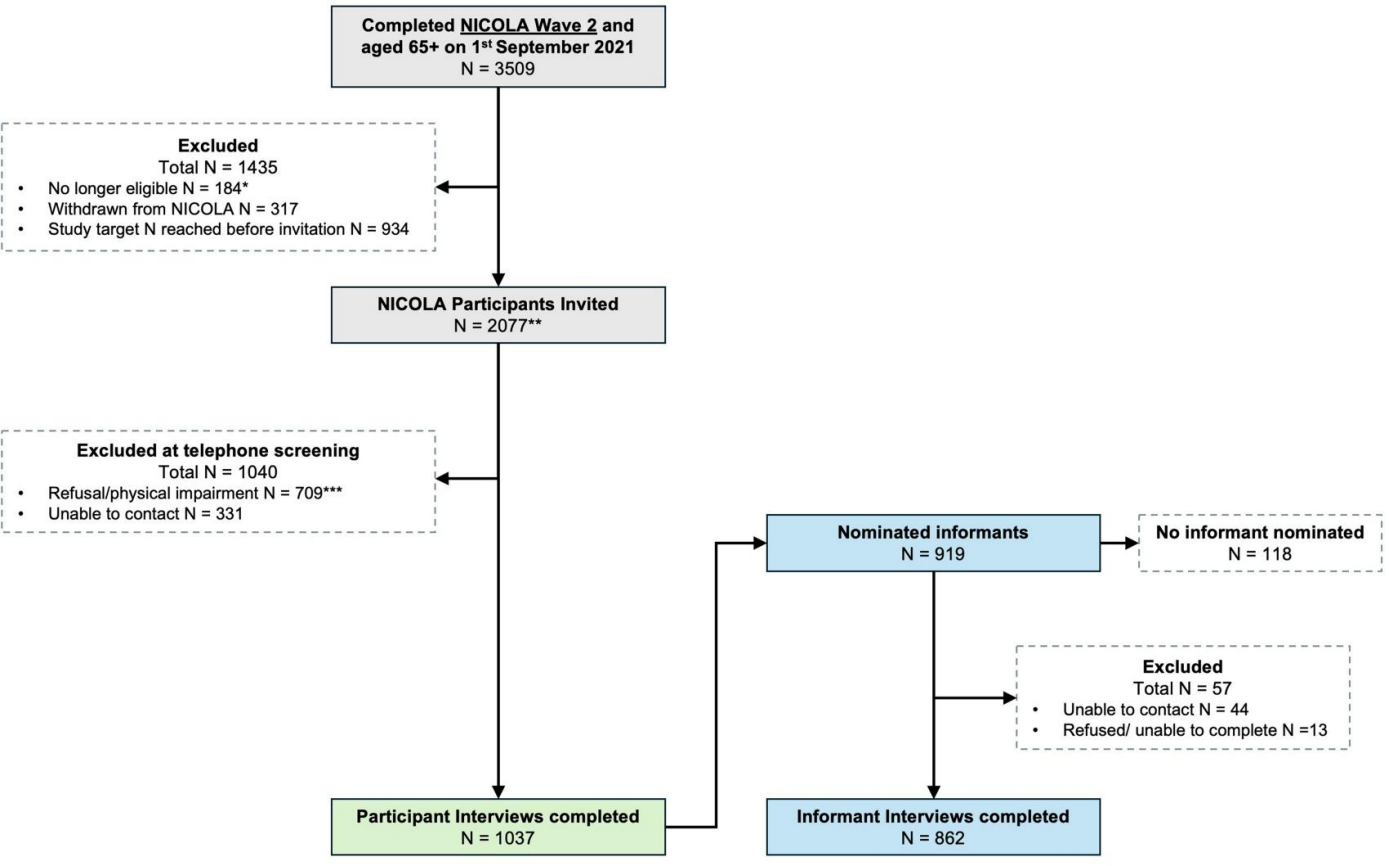
Flow diagram of respondent and informant selection through NICOLA-HCAP. *Comprises participants who passed away, emigrated, or moved into residential care since 1 September 2012 and participants who took part in the NICOLA-HCAP pilot study. **N includes three NICOLA participants who were originally ineligible as they were <65 years old but subsequently aged into eligibility and were allowed to participate at the request of their spouse. ***Those excluded due to physical impairment all reported having severe visual or hearing impairments.

A total of 3509 NICOLA participants met the eligibility criteria (Fig. 1). Originally, 50% of the eligible single-person households (SPHs) and 50% of the eligible multiple-person households (MPHs) were randomly selected to be invited to the study (if both participants in an MPH were eligible, then both were selected for invitation). However, this random sample was exhausted before the target *N* of 1000 was reached. At this point, to increase the recruitment of individuals with lower cognitive function, a targeted selection of the remaining eligible participants with W2 Mini-Mental State Examination (MMSE) scores of ≤26 was conducted. An additional random selection of 50% of the remaining SPHs/MPHs was also conducted. Overall, 2077 eligible NICOLA participants were selected, invited, and contacted for screening. Of those, 709 (21) individuals either refused or were excluded due to significant physical or sensory impairment, while 331 (16%) were not reachable for telephone screening after three attempts. The remaining 1037 individuals were recruited to the study and completed the participant interview, giving a final response rate of 50%. The recruitment process is comprehensively described in Supplementary Section S2.

The response rate was slightly higher in males compared with females (Table 1). Across the age groups, the response rate was highest among those aged 70–74 years and lowest among those aged ≥85 years. The response rate was highest among those with university degrees and lowest among those having no formal qualifications beyond primary school. The response rate was notably lower among those with lower cognitive function at W2. Of the 1037 completed participant interviews, 886 (85%) were conducted in participants’ homes and 151 (15%) in a research centre.

**Table 1:** Response rate of participants across demographic groups.

Group	Invited	Completed interview	
	*N*	*N*	% of invited individuals
Gender			
Male	954	498	52.2
Female	1123	539	48.0
Age at invitation (years)			
65–69	465	245	52.7
70–74	557	305	54.8
75–79	495	244	49.3
80–84	299	141	47.2
≥85	261	102	39.1
Educational attainment^a^			
None/primary school	502	172	34.3
Secondary school (leaving certificate/GCSE/A-level)	909	427	47.0
College level (diploma/certificate)	294	166	56.5
University level (undergraduate/postgraduate degree)	372	272	73.1
W2 MMSE score			
>26	1695	919	54.2
≤26	354	110	31.1

GCSE, General Certificate of Secondary Education.

a Educational attainment measured at NICOLA W2.

Of the 1037 NICOLA-HCAP participants, 862 informant interviews were completed, giving an informant response rate of 83.1% (Fig. 1). One hundred and eighteen participants (11.4%) did not nominate an informant, 44 nominated informants (4.2%) could not be contacted, and 13 nominated informants (1.3%) either refused or were unable to complete the interview when contacted. Of the 862 completed informant interviews, 837 (97.1%) were conducted in person or over the phone and 25 (2.9%) were completed as an online questionnaire.

## What has been measured?

NICOLA-HCAP used the same battery of cognitive tests and informant measures as HRS-HCAP and ELSA-HCAP (see Table 2). Cognitive tests were initially selected by HRS based on several criteria, including ease of administration and sensitivity in predicting the dementia diagnosis. The tests measured performance across a range of cognitive domains, including episodic memory, language, executive function, and visuospatial ability (see Supplementary Table S3). A brief measure of depressive symptoms—the 11-item short form of the Centre for Epidemiological Studies Depression Scale (CES-D)—was included to account for the potential influence of depression on test performance. Three tests—MMSE, animal naming, and CES-D (full version)—were previously administered in NICOLA W1 (the MMSE was also administered in W2). Information from a family member or friend on the nature and severity of the cognitive change and any impairment in activities of daily living is an important part of the clinical evaluation for dementia [22]. The HCAP informant interview incorporated several validated measures designed to capture this information.

**Table 2:** The cognitive battery and informant interview content of the NICOLA-HCAP sub-study versus the core NICOLA waves.

Measure	Measured in NICOLA-HCAP	Measured in core NICOLA study
**Participant interview**		
MMSE	✓	✓
HRS-TICS	✓	X
CERAD Word List Recall (immediate recall)	✓	**X^a^**
Verbal Fluency (Animal Naming)	✓	✓
Letter Cancellation	✓	X
Backward Counting	✓	X
CSI-D	✓	X
CERAD Word List Recall (delayed recall)	✓	**X^a^**
Brave Man (Immediate)	✓	X
Logical Memory (Immediate)	✓	X
CERAD Word List Recognition	✓	X
Constructional Praxis (Immediate)	✓	X
Symbol Digit Modalities Test	✓	X
Constructional Praxis (Delayed)	✓	X
Brave Man (Delayed)	✓	X
Logical Memory (Delayed)	✓	X
Logical Memory (Recognition)	✓	X
Number Series	✓	X
Raven’s Matrices	✓	X
Trail Making A & B	✓	X
CES-D 11	✓	**✓^b^**
**Informant interview**		
Jorm IQCODE	✓	X
Blessed Part 2	✓	X
HRS Activities Questionnaire	✓	X
CSI-D—Cognitive Activities	✓	X
10/66—Informant	✓	X
Blessed Part 1	✓	X

HRS-TICS, Health and Retirement Study—Telephone Interview for Cognitive Status; CERAD, Consortium to Establish a Registry for Alzheimer’s Disease; CSI-D, Community Screening Instrument for Dementia; CES-D, Centre for Epidemiological Studies Depression Scale; IQCODE, Informant Questionnaire on Cognitive Decline in the Elderly.

✓= Measure was included.

X = Measure was not included.

a A similar word-list recall task was administered in W1 using different word stimuli and a different administration protocol.

b A longer 20-item version of this measure was used in NICOLA W1.

### Data quality

HCAP interviews were audio-recorded to assist with quality checking. Following each participant interview, fieldwork staff would listen to the audio recordings to ensure the correct recording of responses for certain verbal tests (Consortium to Establish a Registry for Alzheimer’s Disease Word List Recall, Animal Naming, Backward Counting, Brave Man and Logical Memory). At the end of the fieldwork period, a random sub-sample of 50% of the informant interviews were selected for quality checking, which again involved listening to recordings to ensure the correct recording of responses. Data cleaning involved screening raw data for missing or impossible values. Any missing item responses were checked by using the available audio recordings to ascertain whether a response was given by the participant.

## What has it found? Key findings and publications

The NICOLA-HCAP sub-study provides a detailed neuropsychological assessment of a random sample of participants aged ≥65 years that can be extrapolated to the rest of the NICOLA population and, by extension, to those who are living in the community in NI. Table 3 summarizes the respondents’ characteristics, where 52% were female, 17% had no formal qualifications, and most (67%) were married or cohabiting. Women tended to have higher education and were more likely to be separated, divorced, or widowed. Smoking prevalence was higher among men (11%) than women (7%), with men also three times more likely to report excessive alcohol consumption. In terms of clinical measures, 31% of women screened positive for risk of depression compared with 24% of men. No gender differences were observed in functional impairment or self-reported memory concerns, although, notably, around half of the sample reported memory concerns.

**Table 3:** Characteristics of NICOLA-HCAP participants.

Characteristic [mean (SD)/*n* (%)]	Whole sample (*n* = 1037)	Men (*n* = 498)	Women (*n* = 539)	*P* ^a^
**Demographics**				
Age at HCAP interview (years)	74.0 (6.5)	74.0 (6.4)	74.0 (6.7)	.952
Marital status^b^				<.001
Married/co-habiting	692 (66.8)	375 (75.5)	317 (58.8)	
Single (never married)	64 (6.2)	23 (4.6)	41 (7.6)	
Separated/divorced/widowed	280 (27.0)	99 (19.9)	181 (33.6)	
Geography^b^				.586
Urban	622 (60.0)	303 (60.8)	319 (59.2)	
Rural^c^	415 (40.0)	195 (39.2)	220 (40.8)	
**Socio-economic status**				
Educational attainment				<.001
None/primary school	172 (16.6)	96 (19.3)	76 (14.1)	
Secondary school (leaving certificate/GCSE/A-level)	427 (41.2)	186 (37.3)	241 (44.7)	
College level (diploma/certificate)	166 (16.0)	65 (13.1)	101 (18.7)	
Neighbourhood deprivation quintile^d^				.807
1 (most deprived)	120 (11.6)	56 (11.2)	64 (11.9)	
2	179 (17.3)	82 (16.5)	97 (18.0)	
3	215 (20.7)	99 (19.9)	116 (21.5)	
4	223 (21.5)	113 (22.7)	110 (20.4)	
5 (least deprived)	300 (28.9)	148 (29.7)	152 (28.2)	
**Lifestyle factors**				
Smoking^b^				<.001
Never	520 (51.3)	211 (43.9)	309 (58.1)	
No, stopped	406 (40.1)	218 (45.3)	188 (35.3)	
Yes, smoking at present time	87 (8.6)	52 (10.8)	35 (6.6)	
Alcohol consumption^b^ (>14 units of per week^e^)	118 (19.5)	86 (30.0)	32 (10.1)	<.001
**Clinical and functional measures**				
Risk of Depression (CES-D-short form^f^)	284 (27.4)	119 (23.9)	165 (30.6)	.019
Impaired on at least one instrumental activity of daily living^b^	90 (8.71)	35 (7.1)	55 (10.2)	.302
Impaired on at least one activity of daily living^b**^	110 (14.1)	49 (13.9)	61 (14.3)	.724
Self-reported memory concern	512 (49.4)	238 (47.8)	274 (50.8)	.359

GCSE, General Certificate of Secondary Education; CES-D, Centre for Epidemiological Studies Depression Scale.

Demographic variables were all measured at W2 of the main NICOLA study, with the exception of CES-D short form from the HCAP fieldwork, geography, and neighbourhood deprivation, which were derived based on participant postcodes at the start of the HCAP fieldwork.

a *P* value of chi-square test for associations between categorical variables and independent samples *t*-test for continuous variables.

b Missing data: marital status *n* = 1; geography *n* = 1; smoking *n* = 24; alcohol consumption *n* = 432; instrumental activities of daily living impairment *n* = 4; activities of daily living impairment *n* = 258.

c Urban/rural classification is based on the Northern Ireland Statistics and Research Agency Statistical Classification and Delineation of Settlements [23].

d Neighbourhood deprivation is based on the Northern Ireland Multiple Deprivation Measure 2017 [24], which ranks geographical areas according to deprivation, from 1 (most deprived) to 890 (least deprived). For the present study, these rankings were categorized into quintiles.

e Excessive alcohol consumption defined as >14 units per week, per the UK national and NHS guidelines.

f Depressive symptoms established by using a threshold of 9 on an 11-item version of the Centre for Epidemiological Studies Depression Scale (CES-D).

Technical issues limited the interview-duration recording to 897 participants, with an average of 58.2 minutes (12.4 SD). The missing-data rates were low, but slightly increased for tests conducted later in the battery, likely due to participant fatigue. The average MMSE score was 28.5 (2.1 SD). A cut-off score of ≤26 was previously recommended to identify cognitive impairment in a highly educated sample [25]; in the present sample, 104 participants (10%) scored below this cut-off. Table 4 reports the raw means of the cognitive tests in the NICOLA-HCAP participant interview by age group. The results are further gender-stratified and presented in Supplementary Table S4a. Full descriptive statistics for all of the NICOLA-HCAP outcome measures for participants and nominated informants are provided in Supplementary Table S4b.

**Table 4:** Descriptive statistics presenting raw means and standard deviations of the cognitive tests in the NICOLA-HCAP respondent interview by age group.

Test	Overall		65–69 years		70–74 years		75–79 years		80–84 years		≥85 years	
	*n* ^a^	Mean (SD)	*N*	Mean (SD)	*n*	Mean (SD)	*n*	Mean (SD)	*n*	Mean (SD)	*n*	Mean (SD)
MMSE	1008	28.5 (2.1)	232	29.1 (1.2)	301	28.9 (1.5)	244	28.5 (1.8)	138	28.1 (1.9)	93	26.2 (3.8)
HRS-TICS	1033	2.8 (0.4)	236	2.9 (0.3)	303	2.9 (0.3)	249	2.8 (0.4)	143	2.7 (0.4)	102	2.6 (0.6)
CERAD Word List												
Immediate Recall	1026	18.6 (4.5)	235	20.3 (4)	302	19.2 (4)	247	18.3 (4.5)	142	17.9 (3.9)	100	14.5 (4.7)
Delayed Recall	1025	5.6 (2.4)	235	6.5 (2.1)	301	5.9 (2.2)	247	5.5 (2.3)	142	4.9 (2.4)	100	3.6 (2.3)
Recognition	1021	18.9 (1.7)	235	19.3 (1.3)	302	19.2 (1.3)	245	18.8 (1.6)	142	18.6 (1.9)	97	17.8 (2.4)
Verbal Fluency (Animal Naming)	1034	19.3 (6.2)	236	21.7 (5.9)	303	20.3 (5.9)	249	18.1 (6)	143	18.6 (5.9)	103	14.9 (5.7)
Letter cancellation correctly marked letters	1008	16.1 (4.9)	234	17.9 (4.6)	298	16.7 (4.3)	246	15.7 (5)	137	15.2 (4.6)	93	11.8 (4.6)
Backward Count	1027	33.5 (10.0)	236	37.1 (10.2)	302	35 (9.4)	248	32.8 (9.4)	142	31.1 (8.9)	99	25.7 (9.4)
CSI-D	1031	4.0 (0.2)	235	4 (0.1)	303	4 (0.1)	248	4 (0.2)	143	4 (0.2)	102	3.9 (0.4)
Brave Man												
Immediate Recall	1031	2.4 (1.1)	236	2.6 (1.1)	303	2.5 (1.1)	249	2.3 (1.1)	143	2.1 (1.1)	100	2 (1.1)
Delayed Recall	1019	1.3 (1.1)	236	1.6 (1.1)	301	1.5 (1.1)	246	1.2 (1)	142	1.1 (1)	94	0.9 (1)
Logical Memory												
Immediate Recall	1028	8.9 (4.1)	236	10.1 (3.9)	303	9.4 (4)	248	8.8 (3.9)	143	8.4 (4.2)	98	5.5 (3.4)
Delayed Recall	1014	6.5 (4.1)	235	7.7 (3.8)	301	7.2 (4.1)	245	6.3 (3.9)	142	5.6 (4)	91	3.2 (3.1)
Recognition	971	11.6 (2.1)	234	12.1 (2)	289	11.8 (2)	239	11.5 (2.2)	136	11.5 (2.1)	73	10.1 (2.1)
Constructional Praxis												
Immediate	1019	9.6 (1.6)	235	10 (1.4)	301	9.7 (1.5)	247	9.5 (1.7)	140	9.5 (1.5)	96	8.9 (2.1)
Delayed	1019	7.8 (2.9)	235	8.8 (2.3)	301	8.4 (2.5)	247	7.7 (2.9)	140	7.2 (2.9)	96	5.2 (3.6)
Symbol Digit Modalities Test	1005	35.2 (11.2)	235	40.7 (9.9)	299	38.2 (9.8)	244	33.2 (10.3)	138	30.9 (9.6)	89	22.5 (9.5)
Number Series	990	536.8 (25.2)	232	542.1 (22)	291	540.4 (24.1)	242	535.9 (23.6)	139	529 (30.8)	86	526.1 (25.1)
Raven’s Matrices	1004	14.1 (2.7)	234	14.9 (2.3)	300	14.5 (2.5)	244	13.9 (2.7)	138	13.6 (2.7)	88	12 (3)
Trail Making Test												
Part A Time (seconds)	996	46.3 (21.7)	235	38.5 (15.3)	297	41.2 (15.8)	243	47.9 (17.3)	137	50.5 (18.2)	84	75.2 (39.1)
Part B Time (seconds)	909	115.2 (53.9)	222	94.5 (47.2)	283	107.1 (49)	221	123 (50.6)	120	130.7 (52.2)	63	168 (64)
CES-D	981	1.9 (2.3)	224	1.7 (2.2)	292	1.8 (2.3)	239	1.8 (2.1)	137	2.1 (2.4)	89	2.5 (2.6)

HRS-TICS, Health and Retirement Study—Telephone Interview for Cognitive Status; CERAD, Consortium to Establish a Registry for Alzheimer’s Disease; CSI-D, Community Screening Instrument for Dementia; CES-D, Centre for Epidemiological Studies Depression Scale.

a *n* = 3 participants were excluded from above analysis due to missing age data.

After the completion of data collection, NICOLA-HCAP began pre-statistical harmonization with HRS, TILDA, and ELSA. This included meetings to discuss the test administration, data quality, and cleaning to ensure consistency across the studies. An algorithm will be developed to classify participants as having probable dementia or MCI, similarly to HRS [19], creating a valuable resource for future research on dementia risk factors.

The NICOLA-HCAP data are being finalized and will be available for external research. Several studies have used existing cognitive data within NICOLA W1 and W2 to examine the predictors of cognitive health in older age. For example, previous analyses have examined whether cognitive function is associated with retinal microvasculature parameters [26], chronic kidney disease [27], and traumatic conflict exposure [28]. Future research will leverage the more detailed cognitive assessments in NICOLA-HCAP to examine cognitive outcomes across various domains and over time.

## What are the main strengths and weaknesses?

NICOLA-HCAP is directly harmonized with the HCAP sub-studies in HRS, ELSA, and TILDA, using identical measures and closely matched administration and scoring protocols. As such, algorithm-based estimates of the prevalence of dementia and MCI in NI established using NICOLA-HCAP data will be comparable with prevalence estimates in the USA, England, and Republic of Ireland. There is also substantial overlap in the measures used by other national studies in the HCAP network, which provides opportunities for investigation of the reliability of tests used to measure cognitive function across different countries and cultures [29, 30].

Importantly, the response rate for NICOLA-HCAP (50%) was notably lower than those for other comparable HCAP sub-studies (e.g. 79% in HRS [13]; 76% in ELSA [14]). The lower response rate may be partly attributable to the fact that the fieldwork for HCAP was conducted during the COVID-19 pandemic; some eligible individuals refused due to concerns about having a researcher visit them at home. The low response rate was especially pronounced among certain demographics, namely those who were older, had lower educational attainment, and had lower cognitive function at the previous NICOLA wave. HCAP weights will be created partly to account for bias introduced by these patterns of non-response. Furthermore, unlike other HCAP sub-studies, NICOLA-HCAP did not collect data from participants who were in residential care, implying that the study likely failed to capture data from individuals with more severe dementia who are more likely to be living in residential care.

Furthermore, NICOLA-HCAP is currently a cross-sectional study with only one wave of cognitive assessments. Other studies within the HCAP network have conducted second waves, providing richer longitudinal data on cognitive change and dementia incidence. Additional funding is currently being sought to carry out a second wave of HCAP within NICOLA.

## Can I get hold of the data? Where can I find out more?

Researchers can request access to NICOLA-HCAP data by applying to the NICOLA Data Access Committee (https://www.qub.ac.uk/sites/NICOLA/ForResearchers/). The data have been submitted to data repositories and are publicly available via the Dementias Platform UK data portal (https://portal.dementiasplatform.uk/) and the GGA Data platform (https://g2aging.org/home).

## Supplementary Material

dyag029_Supplementary_Data

## Data Availability

The data underlying this article are available via the NICOLA Data Access Committee at https://www.qub.ac.uk/sites/NICOLA/ForResearchers/ and through public repositories including the Dementias Platform UK data portal (https://portal.dementiasplatform.uk/) and the Gateway to Global Aging Data platform (https://g2aging.org/home).
