# Supplementary material for: Cohort Profile Update: The Harmonised Cognitive Assessment Protocol sub-study of the Northern Ireland Cohort for the Longitudinal Study of Ageing (NICOLA-HCAP)
Source: Int J Epidemiol. 2026 Mar 5;55(2):dyag029. doi: 10.1093/ije/dyag029 (PMC13016766; doi:10.1093/ije/dyag029)
Supplement: dyag029_Supplementary_Data [file dyag029_supplementary_data.docx]

**Supplementary material**

**Contents**

[**Supplementary section 1:** Informant characteristics 2](#_Toc221857721)

[**Table S1.** Sample characteristics of the informants in the Northern Ireland Cohort for the Longitudinal Study of Ageing – Harmonised Cognitive Assessment Protocol (NICOLA-HCAP) 2](#_Toc221857722)

[**Supplementary section 2:** The Northern Ireland Cohort for the Longitudinal Study of Ageing – Harmonised Cognitive Assessment Protocol (NICOLA-HCAP) recruitment summary 3](#_Toc221857723)

[**Supplementary section 3:** Cognitive Battery and Informant Interview Content 5](#_Toc221857724)

[**Table S3.** The Northern Ireland Cohort for the Longitudinal Study of Ageing – Harmonised Cognitive Assessment Protocol (NICOLA-HCAP) Cognitive Battery and Informant Interview Content 5](#_Toc221857725)

[**Supplementary section 4:** Raw Cognitive test scores 7](#_Toc221857726)

[**Table S4a.** Descriptive statistics of the cognitive tests in the Northern Ireland Cohort for the Longitudinal Study of Ageing – Harmonised Cognitive Assessment Protocol (NICOLA-HCAP) respondent interview by gender 7](#_Toc221857727)

[**Table S4b**. Descriptive statistics for all the Northern Ireland Cohort for the Longitudinal Study of Ageing – Harmonised Cognitive Assessment Protocol (NICOLA-HCAP) outcome measures 8](#_Toc221857728)

[**References** 9](#_Toc221857729)

# **Supplementary section 1:** Informant characteristics

## **Table S1.** Sample characteristics of the informants in the Northern Ireland Cohort for the Longitudinal Study of Ageing – Harmonised Cognitive Assessment Protocol (NICOLA-HCAP)

| Category | N | % |
| --- | --- | --- |
| Gender |  |  |
| Male | 285 | 33.1 |
| Female | 577 | 66.9 |
| Age |  |  |
| 20-29 | 10 | 1.2 |
| 30-39 | 39 | 4.5 |
| 40-49 | 101 | 11.7 |
| 50-59 | 118 | 13.7 |
| 60-69 | 226 | 26.2 |
| 70-79 | 289 | 33.5 |
| 80+ | 76 | 8.8 |
| Missing | 3 | 0.3 |
| Education |  |  |
| None/primary school | 54 | 6.3 |
| Secondary school | 354 | 41.1 |
| College level | 154 | 17.9 |
| University level | 300 | 34.8 |
| Relationship to participant |  |  |
| Spouse/partner | 475 | 55.1 |
| Child | 248 | 28.8 |
| Sibling | 40 | 4.6 |
| Friend/neighbour | 64 | 7.4 |
| Other | 35 | 4.1 |

# **Supplementary section 2:** The Northern Ireland Cohort for the Longitudinal Study of Ageing – Harmonised Cognitive Assessment Protocol (NICOLA-HCAP) recruitment summary

The selection and screening process for NICOLA-HCAP is summarised in Figure S1. The sampling frame for the HCAP sub-study comprised all surviving community-dwelling participants who completed Wave 2 of the main NICOLA study and were aged 65 or over on the 1^st^ of September 2021. Selection was done at the household level. There were 2856 households that included at least one eligible individual; 912 single person households (SPH; i.e., households where an eligible participant lived alone) and 1944 multiple person households (i.e., households where an eligible participant lived with others). Within an MPH there may have been one or two eligible individuals; if two individuals were eligible, both were selected. In MPHs with two eligible individuals, these were usually spouses, but less commonly may have been other relationships (e.g., siblings). In total, the 2856 households comprised 3509 individuals (912 individuals in SPHs and 2597 individuals in MPHs).

The HCAP sub-study aimed to recruit 1000 participants. Selection for the study was conducted in phases. In Phase 1, half of the eligible SPHs and half of the eligible MPHs were selected; this comprised 1754 individuals (456 individuals in SPHs and 1298 individuals in MPHs). The remaining 1755 eligible individuals were held in reserve. The selected group of 1754 individuals was exhausted before the target sample was achieved, necessitating a second phase of selection. At this time, due to low response rates among individuals with lower cognitive function at Wave 2 (i.e., MMSE score ≤26), a phase of targeted recruitment was conducted (Phase 2a). From the 1755 eligible individuals held in reserve, all individuals with a Wave 2 MMSE score of ≤26 were invited to take part (along with any eligible spouses). This comprised 298 individuals (87 individuals in SPHs and 211 individuals in MPHs). Additionally, half of the remaining reserved SPHs and half of the remaining reserved MPHs were selected (selection Phase 2b). This comprised 733 individuals (185 individuals in SPHs and 548 individuals in MPHs). Overall, across both phases of selection, 2785 individuals were selected.

Following selection, the recruitment process involved two stages. First, a letter of invitation was posted to the address held for each individual. Around a week later, the NICOLA-HCAP study administrator telephoned each participant to conduct telephone screening. If the participant was deemed eligible and was willing to participate, the administrator then scheduled an appointment for them to complete the interview. Throughout the recruitment process, individuals were identified as no longer being eligible, either via contact with participants or their family members or through regular administrative updates to the core NICOLA database. These participants were excluded (reasons for no longer being eligible were having passed away, emigrated or moved to residential care). Furthermore, several of the selected individuals had taken part in the NICOLA-HCAP pilot study, and as such were not invited to take part in the main HCAP study. 317 individuals asked to be withdrawn from the NICOLA study when contacted, and as such were not screened for HCAP. The target sample size for the study was reached before letters of invitation were sent out to all selected individuals; 210 were yet to receive a letter.

Therefore, the total number of eligible individuals who were invited and contacted for telephone screening was 2074. Three additional NICOLA participants who were originally ineligible due to being younger than 65 were allowed to take part in the study at the request of their spouses. Each of these originally ineligible individuals had turned 65 by the time of telephone screening.

#

# **Supplementary section 3:** Cognitive Battery and Informant Interview Content

## **Table S3.** The Northern Ireland Cohort for the Longitudinal Study of Ageing – Harmonised Cognitive Assessment Protocol (NICOLA-HCAP) Cognitive Battery and Informant Interview Content

| Measure | Reference | Scoring | Domain measured |
| --- | --- | --- | --- |
| **Participant Interview** | | | |
| MMSE | (1) | MMSE: Total Score calculated using highest score out of WORLD or Serial 7s (approach used in NICOLA wave 1) | Global cognitive status |
| HRS-TICS | (2) | HRS-TICS: Total Score | Global cognitive status |
| CERAD Word List Recall (immediate recall) | (3) | CERAD Word List Recall Immediate: Total Score | Episodic memory |
| Verbal Fluency (Animal Naming) | (3) | Verbal Fluency: Total Score | Language/fluency |
| Letter Cancellation | (4) | Letter Cancellation: Correctly marked letters (based on letter underlined) | Attention/processing speed |
| Backward Counting | (5) | Backward Count: Total Score | Attention/processing speed |
| CSI-D | (6,7) | CSI-D: Total Score | Global cognitive status |
| CERAD Word List Recall (delayed recall) | (3) | CERAD Word List Recall Delayed: Total Score | Episodic memory |
| Brave Man (Immediate) | (8) | Story Recall Immediate: Brave Man Exact Score (total no. of exact responses) | Episodic memory |
| Logical Memory (Immediate) | (9) | Story Recall Immediate: Logical Memory Exact Score (total no. of exact responses) | Episodic memory |
| CERAD Word List Recognition | (3) | CERAD Word List Recall Recognition: total no. of correct responses | Episodic memory |
| Constructional Praxis (Immediate) | (3, 10) | Constructional Praxis Immediate: Total Score | Visuospatial ability |
| Symbol Digit Modalities Test | (11) | SDMT: Total Score | Attention/processing speed |
| Constructional Praxis (Delayed) | (3, 10) | Constructional Praxis Delayed: Total Score | Episodic memory |
| Brave Man (Delayed) | 8) | Story Recall Delayed: Brave Man Exact Score (total no. of exact responses) | Episodic memory |
| Logical Memory (Delayed) | (9) | Story Recall Delayed: Logical Memory Exact Score (total no. of exact responses) | Episodic memory |
| Logical Memory (Recognition) | (9) | Story Recall Delayed: Logical Memory Recognition Total Score | Episodic memory |
| Number Series | (12) | Number Series: W-score | Executive function |
| Raven’s Matrices | (13) | Raven's Matrices: Total Score | Abstract reasoning |
| Trail Making A & B | (14) | Trail Making Test:  Part A Time (seconds:centiseconds)  Part B Time (seconds:centiseconds; times > 300s recoded as missing) | Executive function/attention/processing speed |
| CES-D | (15,16) | CESD: Total Score | Depressive symptoms |
| **Informant interview** |  |  |  |
| Jorm IQCODE | (17) |  | Everyday cognitive function |
| Blessed Part 2 | (18) |  | Activities of daily living |
| HRS Activities Questionnaire |  |  | Activity engagement |
| CSI-D – Cognitive Activities | (6,7) |  | Everyday cognitive function |
| 10/66 – Informant | (6,7) |  | Activities of daily living |
| Blessed Part 1 | (18) |  | Everyday cognitive function/ activities of daily living |

MMSE = Mini Mental State Examination; HRS-TICS = Health and Retirement Study - Telephone Interview for Cognitive Status; CERAD = Consortium to Establish a Registry for Alzheimer’s Disease; CSI-D = Community Screening Instrument for Dementia; CES-D = Centre for Epidemiological Studies Depression Scale

# **Supplementary section 4:** Raw Cognitive test scores

## **Table S4a.** Descriptive statistics of the cognitive tests in the Northern Ireland Cohort for the Longitudinal Study of Ageing – Harmonised Cognitive Assessment Protocol (NICOLA-HCAP) respondent interview by gender

| Test | Overall | | Male | | Female | |
| --- | --- | --- | --- | --- | --- | --- |
|  | n | mean (sd) | n | mean (sd) | n | mean (sd) |
| MMSE | 1011 | 28.5 (2.1) | 485 | 28.4 (1.9) | 526 | 28.6 (2.2) |
| HRS-TICS | 1036 | 2.8 (0.4) | 498 | 2.8 (0.4) | 538 | 2.8 (0.4) |
| CERAD Word List |  |  |  |  |  |  |
| Immediate Recall | 1029 | 18.6 (4.5) | 494 | 17.7 (4.1) | 535 | 19.4 (4.7) |
| Delayed Recall | 1028 | 5.6 (2.4) | 493 | 5.1 (2.3) | 535 | 6.0 (2.4) |
| Recognition | 1024 | 18.9 (1.7) | 492 | 18.8 (1.7) | 532 | 19.0 (1.6) |
| Verbal Fluency (Animal Naming) | 1037 | 19.3 (6.2) | 498 | 19.5 (6.4) | 539 | 19.1 (6.0) |
| Letter cancellation correctly marked letters | 1011 | 16.1 (4.9) | 484 | 15.3 (4.5) | 527 | 16.8 (5.0) |
| Backward Count | 1030 | 33.5 (10.0) | 496 | 34.8 (10.5) | 534 | 32.3 (9.4) |
| CSI-D | 1034 | 4.0 (0.2) | 496 | 4.0 (0.2) | 538 | 4.0 (0.2) |
| Brave Man |  |  |  |  |  |  |
| Immediate Recall | 1034 | 2.4 (1.1) | 497 | 2.4 (1.1) | 537 | 2.4 (1.1) |
| Delayed Recall | 1022 | 1.3 (1.1) | 494 | 1.2 (1.1) | 528 | 1.4 (1.0) |
| Logical Memory |  |  |  |  |  |  |
| Immediate recall | 1031 | 8.9 (4.1) | 495 | 9.0 (4.0) | 536 | 8.9 (4.2) |
| Delayed recall | 1017 | 6.5 (4.1) | 489 | 6.4 (3.9) | 528 | 6.6 (4.2) |
| Recognition | 974 | 11.6 (2.1) | 471 | 11.5 (2.1) | 503 | 11.8 (2.1) |
| Constructional Praxis |  |  |  |  |  |  |
| Immediate | 1022 | 9.6 (1.6) | 491 | 9.8 (1.5) | 531 | 9.5 (1.7) |
| Delayed | 1022 | 7.8 (2.9) | 491 | 7.9 (2.9) | 531 | 7.8 (2.9) |
| Symbol Digit Modalities Test | 1008 | 35.2 (11.2) | 484 | 33.9 (10.9) | 524 | 36.4 (11.3) |
| Number Series: W-score | 993 | 536.8 (25.2) | 484 | 538.4 (26.6) | 509 | 535.3 (23.6) |
| Raven's Matrices | 1007 | 14.1 (2.7) | 484 | 14.2 (2.7) | 523 | 13.9 (2.7) |
| Trail Making Test |  |  |  |  |  |  |
| Part A Time (seconds) | 999 | 46.3 (21.7) | 478 | 47.6 (21.7) | 521 | 45.1 (21.6) |
| Part B Time (seconds) | 912 | 115.2 (53.9) | 435 | 121.0 (56.1) | 477 | 109.9 (51.2) |
| CES-D | 984 | 1.9 (2.3) | 471 | 1.7 (2.2) | 513 | 2.0 (2.3) |

MMSE = Mini Mental State Examination; HRS-TICS = Health and Retirement Study - Telephone Interview for Cognitive Status; CERAD = Consortium to Establish a Registry for Alzheimer’s Disease; CSI-D = Community Screening Instrument for Dementia; CES-D = Centre for Epidemiological Studies Depression Scale

## **Table S4b**. Descriptive statistics for all the Northern Ireland Cohort for the Longitudinal Study of Ageing – Harmonised Cognitive Assessment Protocol (NICOLA-HCAP) outcome measures

| Test | Valid N | % Missing^a^ | Mean (SD) | Median (IQR) | Min | Max | Possible scale range |
| --- | --- | --- | --- | --- | --- | --- | --- |
| **Participant Interview** |  |  |  |  |  |  |  |
| MMSE | 1011 | 2.4 | 28.5 (2.1) | 29 (28-30) | 13 | 30 | 0-30 |
| HRS-TICS | 1036 | 0.1 | 2.8 (0.4) | 3 (3-3) | 0 | 3 | 0-3 |
| CERAD Word List |  |  |  |  |  |  |  |
| Immediate Recall | 1029 | 0.8 | 18.6 (4.5) | 19 (16-22) | 0 | 30 | 0-30 |
| Delayed Recall | 1028 | 0.9 | 5.6 (2.4) | 6 (4-7) | 0 | 10 | 0-10 |
| Recognition | 1024 | 1.3 | 18.9 (1.7) | 20 (18-20) | 9 | 20 | 0-20 |
| Verbal Fluency (Animal Naming) | 1037 | 0.0 | 19.3 (6.2) | 19 (15-24) | 1 | 39 | 0+ |
| Letter cancellation correctly marked letters | 1011 | 2.5 | 16.1 (4.9) | 16 (13-19) | 2 | 37 | 0-65 |
| Backward Count | 1030 | 0.7 | 33.5 (10.0) | 33 (28-40) | 1 | 76 | 0+ |
| CSI-D | 1034 | 0.3 | 4.0 (0.2) | 4 (4-4) | 2 | 4 | 0-4 |
| Brave Man |  |  |  |  |  |  |  |
| Immediate Recall | 1034 | 0.3 | 2.4 (1.1) | 2 (2-3) | 0 | 6 | 0-6 |
| Delayed Recall | 1022 | 1.4 | 1.3 (1.1) | 1 (0-2) | 0 | 5 | 0-6 |
| Logical Memory |  |  |  |  |  |  |  |
| Immediate recall | 1031 | 0.6 | 8.9 (4.1) | 9 (6-12) | 0 | 22 | 0-25 |
| Delayed recall | 1017 | 1.9 | 6.5 (4.1) | 6 (3-9) | 0 | 20 | 0-25 |
| Recognition | 974 | 6.1 | 11.6 (2.1) | 12 (10-13) | 3 | 15 | 0-15 |
| Constructional Praxis |  |  |  |  |  |  |  |
| Immediate | 1022 | 1.4 | 9.6 (1.6) | 10 (9-11) | 2 | 11 | 0-11 |
| Delayed | 1022 | 1.4 | 7.8 (2.9) | 8 (6-10) | 0 | 11 | 0-11 |
| Symbol Digit Modalities Test | 1008 | 2.8 | 35.2 (11.2) | 36 (27-44) | 4 | 67 | 0-110 |
| Number Series: W-score | 993 | 4.2 | 536.8 (25.2) | 537 (519-549) | 409 | 584 | 409-570/  413-584^b^ |
| Raven's Matrices | 1007 | 2.9 | 14.1 (2.7) | 15 (13-16) | 0 | 17 | 0-17 |
| Trail Making Test |  |  |  |  |  |  |  |
| Part A Time (seconds) | 999 | 3.7 | 46.3 (21.7) | 40.8 (32.4-53.4 | 7.1 | 232.6 | 0-300^c^ |
| Part B Time (seconds) | 912 | 12.1 | 115.2 (53.9) | 100.7 (77.3-138.1 | 33.3 | 299.8 | 0-300^c^ |
| CES-D | 984 | 5.1 | 1.9 (2.3) | 1 (0-3) | 0 | 11 | 0-11 |
| **Informant Interview** |  |  |  |  |  |  |  |
| JORM IQCODE | 689 | 33.6 | 3.1 (0.3) | 3.1 (3.0-3.2) | 2.1 | 5.0 | 1.0-5.0 |
| Blessed Part 2 | 859 | 17.2 | 1.0 (0.2) | 1.0 (1.0-1.0) | 1.0 | 3.7 | 1.0-4.0 |
| CSI-D Informant Score^d^ | 859 | 17.2 | 3.3 (2.9) | 2.5 (1.0-4.5) | 0.0 | 25.0 | 0.0-30.0 |
| Blessed Part 1 | 690 | 33.5 | 0.6 (1.0) | 0.0 (0.0-1.0) | 0.0 | 7.5 | 0.0-8.0 |

MMSE = Mini Mental State Examination; HRS-TICS = Health and Retirement Study - Telephone Interview for Cognitive Status; CERAD = Consortium to Establish a Registry for Alzheimer’s Disease; CSI-D = Community Screening Instrument for Dementia; CES-D = Centre for Epidemiological Studies Depression Scale

^a^ Percentage of total participant sample N = 1037

^b^ Score range varies depending on random assignment to one of two lists of items; see <https://hrs.isr.umich.edu/publications/biblio/5959> for scoring description

^c^ Participants were only allowed up to 300s to complete this test

^d^ CSI-D Informant Score is a slightly adapted version of informant score (‘relscore’) from 10/66 Study (30)

# **References**

1. Folstein MF, Folstein SE, McHugh PR. “Mini-mental state”: a practical method for grading the cognitive state of patients for the clinician. Journal of psychiatric research. 1975;12(3):189-98.
2. Brandt J, Spencer M, Folstein M. The Telephone Interview for Cognitive Status. Cognitive and Behavioral Neurology. 1988;1(2).
3. Morris JC, Heyman A, Mohs RC, Hughes JP, van Belle G, Fillenbaum G, et al. The Consortium to Establish a Registry for Alzheimer's Disease (CERAD). Part I. Clinical and neuropsychological assesment of Alzheimer's disease. Neurology. 1989;39(9):1159.
4. Richards M, Kuh D, Hardy R, Wadsworth M. Lifetime cognitive function and timing of the natural menopause. Neurology. 1999;53(2):308.
5. Lachman ME, Agrigoroaei S, Tun PA, Weaver SL. Monitoring Cognitive Functioning: Psychometric Properties of the Brief Test of Adult Cognition by Telephone. Assessment. 2013;21(4):404-17.
6. Hall KS, Hendrie HC, Brittain HM, Norton JA, et al. The development of a dementia screening interview in two distinct languages. International Journal of Methods in Psychiatric Research. 1993;3(1):1-28.
7. Prince M, Ferri CP, Acosta D, Albanese E, Arizaga R, Dewey M, et al. The protocols for the 10/66 dementia research group population-based research programme. BMC Public Health. 2007;7(1):165.
8. Gfeller JD, Horn GJ. The East Boston Memory Test: A clinical screening measure for memory impairment in the elderly. Journal of Clinical Psychology. 1996;52(2):191-6.
9. Wechsler D. Wechsler Memory Scale - Fourth Edition: Pearson; 2009.
10. Yuspeh RL, Vanderploeg RL, Kershaw DAJ. CERAD Praxis Memory and Recognition in Relation to Other Measures of Memory. The Clinical Neuropsychologist. 1998;12(4):468-74.
11. Smith A. Symbol Digit Modalities Test - Manual. Los Angeles: Western Psychological Service; 1982.
12. Fisher GG, McArdle JJ, McCammon RJ, Sonnega A, Weir DR. New Measures of Fluid Intelligence in the HRS. 2014.
13. Raven JC. Standard Progressive Matrices: Sets A, B, C, D & E: Pearson; 1998.
14. Reitan RM. Trail Making Test: Manual for Administration and Scoring. Tuscon, AZ: Reitan Neuropsychological Laboratory; 1992.
15. Radloff LS. The CES-D Scale: A Self-Report Depression Scale for Research in the General Population. Applied Psychological Measurement. 1977;1(3):385-401.
16. Steffick DE. Documentation of Affective Functioning Measures in the Health and Retirement Study. Ann Arbor, MI: University of Michigan; 2000.
17. Jorm AF. A short form of the Informant Questionnaire on Cognitive Decline in the Elderly (IQCODE): development and cross-validation. Psychological Medicine. 1994;24(1):145-53.
18. Blessed G, Tomlinson BE, Roth M. The Association Between Quantitative Measures of Dementia and of Senile Change in the Cerebral Grey Matter of Elderly Subjects. The British Journal of Psychiatry. 1968;114(512):797-811.
